# Possible association between obesity and periodontitis in patients with Down syndrome

**DOI:** 10.4317/medoral.22311

**Published:** 2018-04-24

**Authors:** Elena Culebras-Atienza, Francisco-Javier Silvestre, Javier Silvestre-Rangil

**Affiliations:** 1 Dental surgeon of the Red Cross Special Patients Dental Clinic, Valencia. Professor of the Master of Hospital Odontology and Special Patients. Department of Stomatology, University of Valencia. Valencia, Spain; 2 Professor of the Department of Stomatology, University of Valencia. Valencia, Spain; 3 Associate Professor of the Department of Stomatology, University of Valencia. Valencia, Spain

## Abstract

**Background:**

The present study was carried out to evaluate the possible association between obesity and periodontitis in patients with DS, and to explore which measure of obesity is most closely correlated to periodontitis.

**Material and Methods:**

A prospective observational study was made to determine whether obesity is related to periodontal disease in patients with DS. The anthropometric variables were body height and weight, which were used to calculate BMI and stratify the patients into three categories: < 25(normal weight), 25-29.9 (overweight) and ≥ 30.0 kg/m2 (obese). Waist circumference and hip circumference in turn was recorded as the greatest circumference at the level of the buttocks, while the waist/hip ratio (WHR) was calculated. Periodontal evaluation was made of all teeth recording the plaque index (PI), pocket depth (PD), clinical attachment level (CAL) and the gingival index. We generated a multivariate linear regression model to examine the relationship between PD and the frequency of tooth brushing, gender, BMI, WHI, WHR, age and PI.

**Results:**

Significant positive correlations were observed among the anthropometric parameters BMI, WHR, WHI and among the periodontal parameters PI, PD, CAL and GI. The only positive correlation between the anthropometric and periodontal parameters corresponded to WHR. Upon closer examination, the distribution of WHR was seen to differ according to gender. Among the women, the correlation between WHR and the periodontal variables decreased to nonsignificant levels. In contrast, among the males the correlation remained significant and even increased. In a multivariate linear regression model, the coefficients relating PD to PI, WHR and age were positive and significant in all cases.

**Conclusions:**

Our results suggest that there may indeed be an association between obesity and periodontitis in male patients with DS. Also, we found a clear correlation with WHR, which was considered to be the ideal adiposity indicator in this context.

** Key words:**Down syndrome, periodontal disease, obesity.

## Introduction

Down syndrome (DS) or trisomy 21 is the most frequent form of mental retardation of genetic origin. It is the most prevalent and best known chromosomopathy, and is associated to multiple comorbidities, a characteristic phenotype and an intellectual defect of variable severity. The survival of patients with DS has improved considerably over the last decades. Medical control of the systemic disorders and progressive social integration in turn have resulted in an increased demand for dental care in this patient population. In this regard, patients with DS present a series of distinctive oral features such as quantitative and qualitative dental anomalies, delayed eruption, higher prevalence of untreated cavities, malocclusions, and a high prevalence of periodontitis (1).

Periodontitis is an inflammatory disease of the tooth supporting tissues, namely the gums, bone and periodontal ligament. It is regarded as the result of an imbalance in the interaction between the host immune system and the microbiota of the marginal dental plaque that colonizes the gingival sulcus. Periodontitis is of multifactorial origin and is produced by the germs present in dental plaque, though other parameters can contribute to its development, such as genetic factors, environmental conditions and also systemic disorders such as diabetes mellitus, immunosuppression, or obesity. Any uncontrolled inflammatory process affecting the tooth supporting tissues can favor development of the disease (2).

Overweight and obesity are characterized by the excessive accumulation of adipose tissue that can have harmful effects upon health. Adipose tissue produces abundant cytokines and adipokines or adipocytokines (3), some of which act locally while others are released into the bloodstream and affect different body organs and systems (4). Some studies (5,6) have reported an association between overweight/obesity and periodontitis. The first study associating obesity to periodontal disease was published in 1977 by Perlstein *et al.* (7), who found bone reabsorption to be greater in obese Zucker rats than in non-obese rats. Other authors have found no direct association, however (8).

In many studies, the parameters used to asses abdominal obesity, such as waist circumference (WC) and the waist/hip ratio (WHR), have been found to be more closely related to periodontal disease than the body mass index (BMI), which is of little help in characterizing the distribution of body fat (9).

The present study was carried out to evaluate the possible association between obesity and periodontitis in patients with DS, and to explore which measure of obesity is most closely correlated to periodontitis.

## Material and Methods

-Study design and population

A prospective observational study was made to determine whether obesity is related to periodontal disease in patients with DS over 18 years of age.

We included patients with DS seen in the Red Cross Special Patients Dental Clinic (Valencia, Spain) for dental control or treatment between 2013 and 2015. The included patients were between 18-54 years of age and had at least 10 teeth in the mouth. Informed consent to participation was obtained from their legal representatives. Patients who had received periodontal treatment within the 6 months previous to this study were excluded, as were individuals wearing orthodontic appliances, smokers, patients receiving bone metabolism-altering drugs, diabetic subjects and patients with other systemic inflammatory disorders. The study sample comprised 90 adults with DS (54 males and 36 females), with a mean age of 29 years.

All the buccodental evaluations were made by the same examiner calibrated before the study as indicated in the epidemiological surveys manual of the World Health Organization (WHO), in order to ensure the reliability and validity of the results.

Measurement error was assessed based on the Dahlberg formula, repeating the measurements of the periodontal variables in 10% of the patients. The results obtained are shown in  Table 1.

**Table 1:**
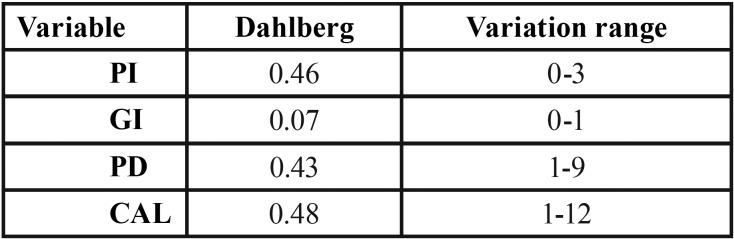
Dahlberg’s error of the periodontal variables.

A dental questionnaire was administered to the parents or tutors, compiling data referred to the anamnesis and oral hygiene habits. Patient dental exploration was then carried out, using a number 5 flat intraoral mirror and a PCP-12 periodontal probe (Hu-Friedy®). In uncooperative patients, the assistant staff resorted to control of the head and hands in order to facilitate the exploration. A customized cheek retractor consisting of the handle of an alginate mixer covered with dressing and sealing tape was applied to one side of the mouth while the other side was explored.

-Study variables

The anthropometric variables were body height (in cm) and weight (in kg), which were used to calculate BMI (kg/m2) and stratify the patients into three categories according to the WHO classification: < 25 (normal weight), 25-29.9 (overweight) and ≥ 30.0 kg/m2 (obese). Each group was formed by 30 patients with DS. The normal weight group was formed by 21 male and 9 female patients. Ages in this group ranged from 18 to 54 years old, with a mean age of 27,17 years. The overweight group was composed by 20 male and 10 female patients with ages ranging from 18 to 49 years old, with a mean age of 32,37 years. And the obesity group gathered 13 male and 17 female patients, ages ranging from 18 to 47 years old, with a mean age of 30,67 years.

Waist circumference (in cm) was measured at umbilical level, and was taken to be an indicator of the amount of visceral adipose tissue that is most active and secretes the largest amounts of cytokines and hormones. Hip circumference in turn was recorded as the greatest circumference (in cm) at the level of the buttocks, while the waist/hip ratio (WHR) was calculated by dividing waist circumference by hip circumference, and was regarded as a measure of visceral fat. A WHR of over 0.9 in males and over 0.8 in females is associated to increased health problems. The gender difference is due to skeletal differences and differences in body fat distribution between males and females.

We decided to use these anthropometric variables because they are simple and inexpensive measures for estimating adipose tissue mass, percentage body fat and body fat distribution, and can be obtained outside the laboratory setting. We also calculated the percentage waist-height index (WHI), calculated by dividing waist circumference by body height (both in cm), and regarded as either adequate (<50%) or high (>50%).

Periodontal evaluation was made of all teeth in the mouth except the third molars, root fragments and teeth not fully erupted (i.e., teeth in which the occlusal surfaces and incisal margins did not reach the occlusal plane. The plaque index (PI) was recorded based on the scale of Silness and Löe. A PCP-12 periodontal probe (Hu-Friedy®) was used, recording the variables in 6 localizations of each tooth. We measured pocket depth (PD), clinical attachment level (CAL) and the gingival index (GI), which indicates the presence of periodontal inflammatory activity based on the Lindhe index, recording the presence or absence of bleeding at each point after waiting for 15-30 seconds to obtain the reading.

-Statistical analysis

The intra-examiner error of the method was estimated using the Dahlberg formula for assessing continuous variables. According to this formula, the closer the mean and standard deviation are to zero, the greater the reproducibility of the measurement process.

Linear correlation models were developed to explore the relationship between the different explanatory variables and their possible interactions. Fitting of the model was measured based on the usual statistics (R2). Statistical significance was considered for *p* ≤ 0.05 in all tests. The R version 3.0.2. statistical package was used throughout.

## Results

The study sample consisted of 90 patients with DS (54 males and 36 females), with a mean age of 29 years (range 18-54).

The mean PI was similar in all three groups: 2.25±0.19 in the patients with normal weight, 2.27±0.19 in the overweight, and 2.26±0.20 in the obese individuals. The Kruskal-Wallis nonparametric test revealed no significant differences in the distribution of the variable of interest among the three groups defined according to BMI (*p*=0.993); no effect of BMI upon PI can therefore be assumed ( Table 1).

The mean percentage locations where bleeding upon probing was observed in the patients of normal weight was 42.19±7.49, versus 47.58±8.92 in the overweight group and 45.70±7.27 in the obese subjects. The Kruskal-Wallis nonparametric test revealed no significant differences in the distribution of the variable of interest among the three groups (*p*=0.67); no effect of BMI upon GI can therefore be assumed ( Table 1).

The mean PD in the patients of normal weight was 2.59 ± 0.15, versus 2.86 ± 0.22 in the overweight group and 2.57 ± 0.22 in the obese subjects. The Kruskal-Wallis nonparametric test revealed no significant differences in the distribution of the variable of interest among the three groups (*p*=0.07); no effect of BMI upon PD can therefore be assumed ( Table 1, Table 2).

**Table 2:**
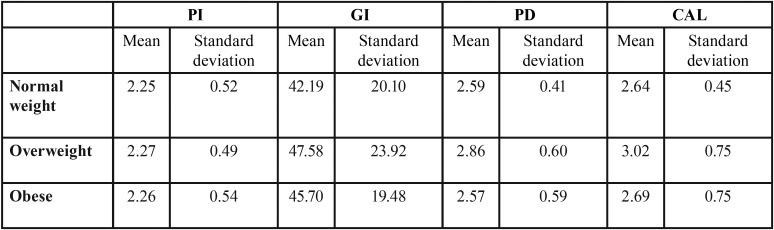
Plaque index, gingival index, pocket depth and clinical attachment level according to body mass index group.

The mean CAL in the patients of normal weight was 2.64 ± 0.17, versus 3.02 ± 0.28 in the overweight group and 2.69 ± 0.28 in the obese subjects. The Kruskal-Wallis nonparametric test revealed no significant differences in the distribution of the variable of interest among the three groups (*p*=0.086); no effect of BMI upon CAL can therefore be assumed ( Table 1, Table 2).

**Figure 1:**
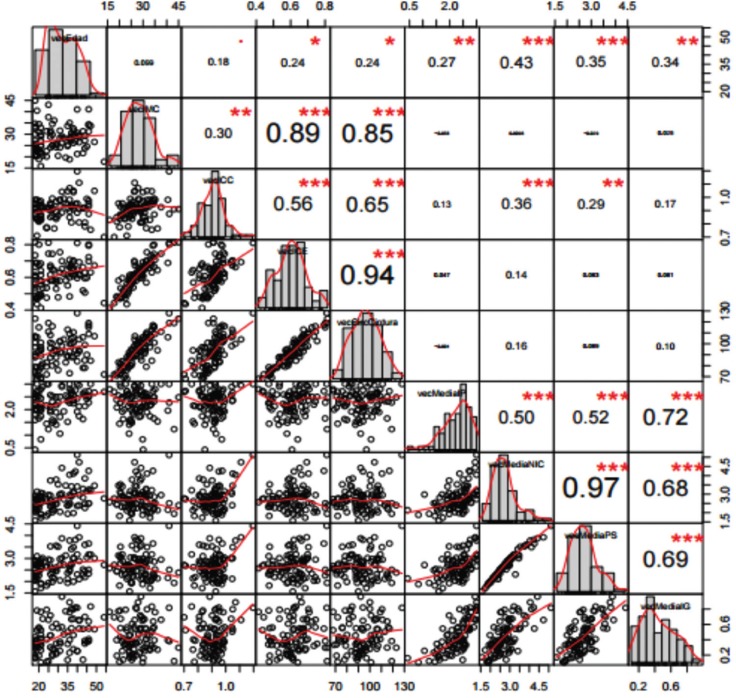
Correlation among anthropometric parameters BMI, WHR, WHI (rows and columns 2 to 4) and periodontal parameters PI, PD, CAL and GI (rows and columns 5 to 8).

Significant positive correlations were observed among the anthropometric parameters BMI, WHR, WHI (rows and columns 2 to 4 in Fig. 1) and among the periodontal parameters PI, PD, CAL and GI (rows and columns 5 to 8 in Fig. 1). The variable age showed a significant positive correlation to all the anthropometric and periodontal parameters. The only positive correlation between the anthropometric and periodontal parameters did not correspond to BMI but to WHR. Upon closer examination, the distribution of WHR was seen to differ according to gender.

**Figure 2:**
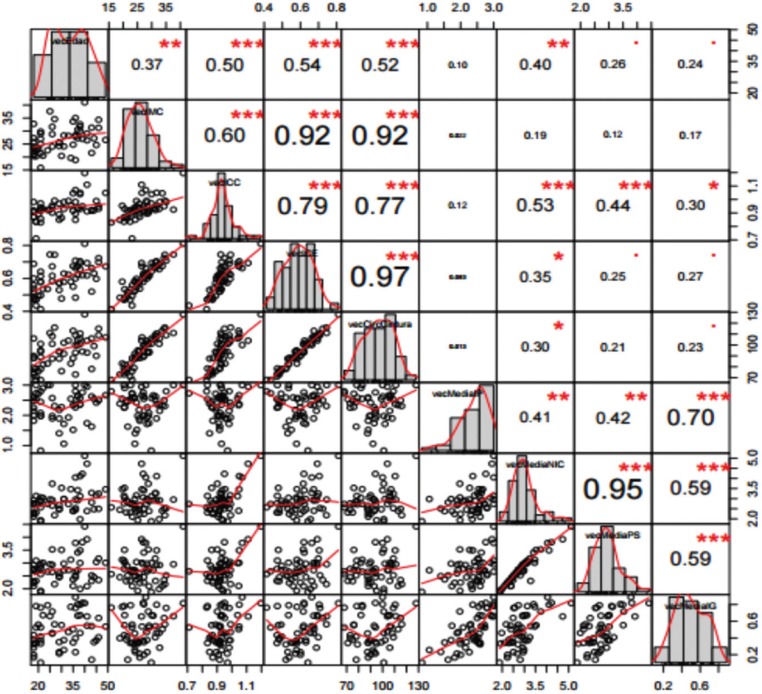
Correlation between WHR and the periodontal variables in women.

**Figure 3:**
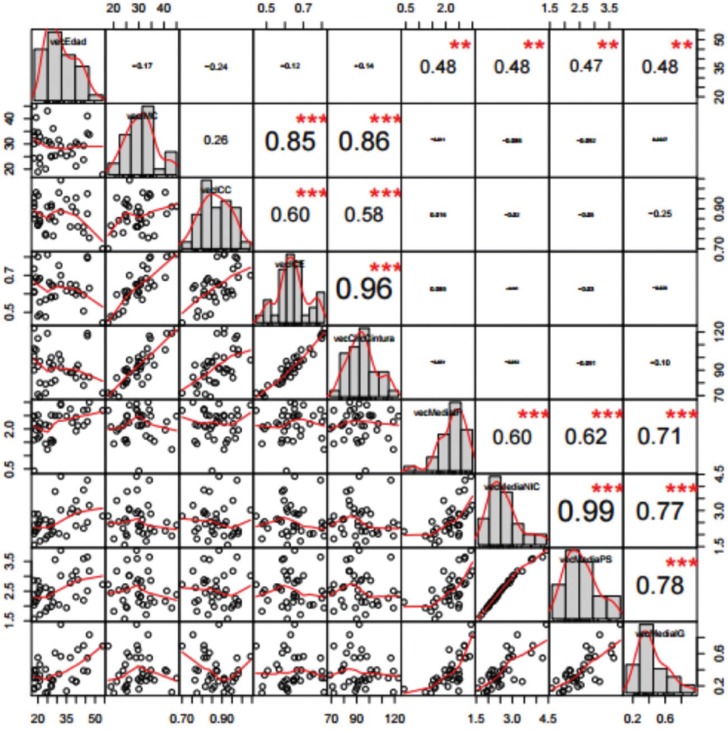
Correlation between WHR and the periodontal variables in men.

Among the women, the correlation between WHR and the periodontal variables decreased to nonsignificant levels. In contrast, among the males the correlation remained significant and even increased (Figs. 2,3).

With the aim of determining which variables influence PD and CAL, we generated a multivariate linear regression model to examine the relationship between PD and the frequency of tooth brushing, gender, BMI, WHI, WHR, age and PI. The coefficients relating PD to PI, WHR and age were positive and significant in all cases. An increase in these variables thus results in an increase in PD among the patients. In any case, the R2 of the model was 0.35; there consequently may be other variables that have not been taken into account, and which can influence PD. With regard to the relationship between gender and WHR, the effect of the latter proved significant in males but not in females. All the coefficients, with the exception of WHR referred to female gender, were positive and significant. An increase in value thus results in an increase in mean PD. Nevertheless, the R2 of the model was 0.36; there consequently may be other variables that have not been taken into account, and which can influence PD. Due to the strong correlation between CAL and PD, similar results were obtained in the study with CAL ( Table 2, Table 3).

**Table 3:**
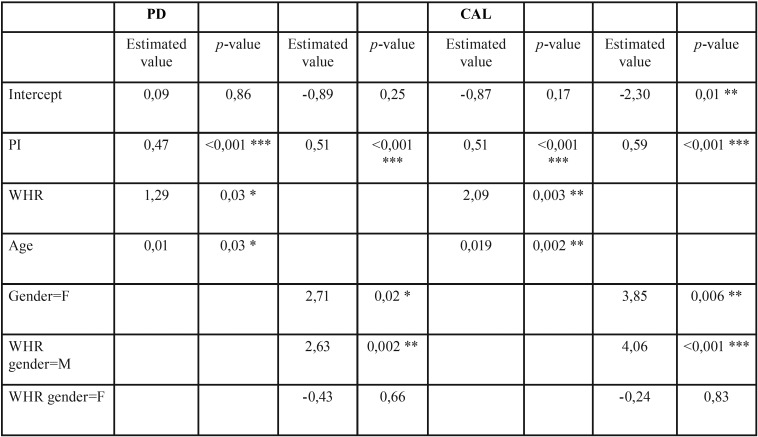
Results of the multivariate linear regression model for pocket depth and clinical attachment level.

## Discussion

Obesity has been has been regarded as a risk factor for many chronic disorders such as hypertension, type 2 diabetes, dyslipidemia and arteriosclerosis. Periodontitis is one of the most common chronic diseases in the world, and has moreover been associated to an increased risk of cardiovascular disease and diabetes mellitus. Obesity in turn could be associated to an alteration in immune response and to an increase in infectious disease such as periodontitis (10). An association between obesity and periodontitis therefore would have important health implications.

The association between obesity and periodontitis remains subject to controversy. Many studies (5,6) have reported a correlation between overweight/obesity and periodontitis. However, while some studies describe a positive association, others have reported only a moderate association (9,11), and some have found no relationship between the two conditions (12).

The adverse impact of obesity upon the periodontium could be mediated by proinflammatory cytokines such as interleukins (IL-1, IL-6 and TNF-α), and an inverse relationship has been reported with antioxidants (13). Genco *et al.* likewise came to the conclusion that obesity is associated to high plasma concentrations of TNF-α and its soluble receptors, which in turn can lead to a hyperinflammatory state with an increase in the risk of periodontal disease and greater gingival inflammation (14).

However, data are lacking on the association between obesity and periodontitis in adults with DS. Periodontal disease is a common oral health problem in such individuals, with a prevalence of 58%-96% in patients under 35 years of age. This study was therefore carried out with the aim of contributing further evidence on the association between obesity and periodontitis in adults with DS.

We found the only positive correlation between the parameters related to obesity, PD and CAL to be WHR, with no correlation to BMI, WHI or WC. An increasing number of authors question the use of BMI as an indicator of obesity, since it does not take into account the size of the individual or whether body weight corresponds to adipose tissue or muscle. The validity of BMI as a measure of obesity decreases with advancing age (11), as a result of changes in body composition, and other parameters such as WC and WHR are regarded as more valid obesity indicators. The accumulation of abdominal visceral adipose tissue (abdominal obesity) (WHR ≥ 0.8 in females and ≥ 0.9 in males) is associated to a greater risk of health problems than the accumulation of subcutaneous adipose tissue, independently of the BMI (15). Our data reflect a similar pattern referred to the association to periodontal disease. Some authors consider WHI to be a better indicator than BMI or WHR in identifying metabolic risk in subjects with ideal body weight, as well as in overweight individuals (16).

Saito *et al.*, found WHR to be correlated to periodontitis independently of BMI (5). Many studies have described an association between periodontitis and central obesity indicators, including WC and WHR (6,9,17,18), though others have been unable to confirm this association (12). In many studies, measures of abdominal obesity such as WC and WHR appear more closely correlated to periodontal disease than BMI (9,18). The association appears to be more consistent referred to visceral adipose tissue than to general adiposity – suggesting that the parameters referred to the accumulation of visceral fat could be more closely associated to periodontitis than BMI. This is concordant with the fact that proinflammatory adipocytokines such as TNF-α and IL-6 are initially produced by the abdominal adipose tissue. Different mechanisms have been implicated in the increase in TNF-α and could contribute to the development of periodontitis. The stimulation of osteoclast formation and the host response to periodontal pathogens produced by TNF-α induce alveolar bone destruction and participate in connective tissue destruction. The association between IL-6 and periodontitis is not clear, due to the pro- and antiinflammatory actions of this interleukin (19).

On examining the variable WHR more closely, we found its distribution to differ according to gender. In women, the correlation between WHR and PD and CAL decreased and proved nonsignificant. In contrast, in men the correlation remained significant and even increased. These results are consistent with those of other studies (6). The topographic distribution of fat can play an important role in the host immune response, and the accumulation of visceral fat can result in an increase in glucose and lipid concentrations, as well as increased insulin resistance. The biology of oral health differs greatly between men and women, with sexual dimorphism in the susceptibility to periodontitis (20).

The sex hormones have profound effects upon a number of immune parameters that regulate both the amplification and resolution of inflammation. There is strong evidence of sexual dimorphism in both innate and acquired immune function. The literature suggests that the hormone- and X chromosome-linked differences in immune response contribute significantly to the relationship between gender and disease (21). Periodontal lesions and infections have been associated to greater concentrations of inflammatory cytokines, including IL-1β and TNF-α, in men than in women. The greater innate immune response in males compared with females could contribute to the observed gender differences in the risk of developing periodontal disease. Another important factor characterizing the gender differences in humoral immunity is the fact that females show increased B lymphocyte activation and antibody production in response to antigens compared with males. In this regard, a more effective humoral response in women would afford greater protection against periodontal pathogens. The balance between modulation of the host immune response and regulation of the resolution of inflammation ultimately determines the gender differences referred to periodontal disease. However, it is still not clear whether inflammation lies at the root of such gender differences. Furthermore, differences in gene regulation – particularly referred to genes related to the sex hormones – may contribute to sexual dimorphism in the severity of periodontal disease (20). It is also possible that WHR is not a good obesity indicator in women, since females do not tend to accumulate fat at waist level but in the region of the hips as a result of estrogen activity, and because lipolytic activity is lower in the hips than in other zones were adipose tissue accumulates and in the region of the hips in males.

A limitation of our study is its cross-sectional design, which makes it difficult to establish the direction of causality and limits the definition of time relationships, i.e., by simultaneously evaluating anthropometric parameters and periodontal conditions it is not clear whether obesity effectively precedes periodontitis or not. A study of incident cases would be more appropriate for clarifying this association, since prevalent cases could underestimate the magnitude of the association and give rise to partial causal effects in the variables that moreover change over time (22). Keller *et al.* carried out a systematic review to analyze the association over time between obesity and periodontitis, and the way in which changes in body weight can affect the development of periodontitis. The review included 8 longitudinal studies, of which three showed a direct association between obesity at the start of the study and the development of periodontitis. Likewise, two of the studies reported a direct correlation between overweight and the development of periodontal disease (23).

Other confounding factors possibly might not have been taken into account in our study, such as physical exercise and eating habits – both of which are related to obesity and periodontal health. Regular physical exercise improves health in general, as well as quality of life, and is inversely correlated to non-transmissible chronic disorders such as cardiovascular disease, hypertension, type 2 diabetes, osteoporosis, obesity, colon cancer, anxiety and depression (24). Some authors have suggested that people who engage in physical exercise have a lesser risk of suffering periodontitis (25), though few studies have investigated this relationship to date. Nutritional status historically has been linked to periodontal disease through scurvy, though additional associations have been demonstrated between nutrition and periodontitis, including the action of vitamins C and E, and carotenoids, as antioxidants in the diet (26). Studies have also been made of the relationship between periodontitis and the protective effects of antioxidant nutrients, due to the association between periodontal disease and an increased production of reactive oxygen species that cause damage to cells and tissues (27). Recent investigations point to a negative correlation between the intake of antioxidant vitamins in the diet and the progression of periodontal disease in Japanese adults (28). Zare *et al.* in turn suggest that dietetic counseling can improve eating habits and therefore the antioxidant status of patients with chronic periodontitis (29). However, the role of dietetic interventions in increasing the intake of foods rich in antioxidants has not been studied in depth in patients with chronic periodontitis, and the relationship between food components and periodontitis is not clear. Further studies are therefore needed to clarify this association.

Some studies have also suggested the implication of psychosocial factors in the association between obesity and periodontitis. There is growing evidence in the recent literature that metabolic syndrome can be triggered and/or exacerbated by adverse social factors, as well as by certain psychological disorders and behaviors, and that metabolic syndrome and obesity may be related to social factors such as dissatisfaction with personal physical appearance, self-esteem, depression and stress (30).

Our results suggest that there may indeed be an association between obesity and periodontitis in male patients with DS. Also, we found a clear correlation with WHR, which was considered to be the ideal adiposity indicator in this context. Due to the different adipose tissue distribution characterizing the female sex, the correlation was not so apparent in women.

Thus, in patients with DS, and because of their increased vulnerability to periodontitis, it is necessary to implement preventive programs and adopt measures to ensure correct oral hygiene, either on an autonomous basis or assisted by the patient caregivers.
